# Thermogenic effect of meltdown RTD™ energy drink in young healthy women: a double blind, cross-over design study

**DOI:** 10.1186/1476-511X-8-57

**Published:** 2009-12-17

**Authors:** Stefanie L Rashti, Nicholas A Ratamess, Jie Kang, Avery D Faigenbaum, Aristomen Chilakos, Jay R Hoffman

**Affiliations:** 1 The College of New Jersey, Ewing, NJ 08628-0718, USA

## Abstract

**Background:**

The purpose of this study was to examine the acute metabolic effects of a high-energy drink in healthy, physically-active women.

**Methods:**

Ten women (20.4 ± 0.70 y; 166.9 ± 7.2 cm; 67.0 ± 7.0 kg; 29.6 ± 6.5% body fat) underwent two testing sessions administered in a randomized and double-blind fashion. Subjects reported to the laboratory in a 3-hr post-absorptive state and were provided either 140 ml of the high-energy drink (SUP; commercially marketed as Meltdown RTD™) or placebo (P). Subjects consumed two 70 ml doses of SUP or P, separated by 30 min and rested in a semi-recumbent position for 3 hours. Resting oxygen consumption (VO_2_) and heart rate (HR) were determined every 5 min during the first 30 min and every 10 min during the next 150 min. Blood pressure (BP) was determined every 15 min during the first 30 min and every 30 min thereafter. Area under the curve (AUC) analysis was computed for VO_2_, whereas a 3-hour average and hourly averages were calculated for respiratory quotient (RQ), total kcal, HR, BP, and profile of mood states (POMS).

**Results:**

AUC analysis revealed a 10.8% difference (p = 0.03) in VO_2 _between SUP and P. No difference in VO_2 _was seen between the groups in the first hour, but VO_2 _in SUP was significantly greater than P in the second (13.9%, p = 0.01) and third hours (11.9%, p = 0.03). A difference (p = 0.03) in energy expenditure was seen between SUP (1.09 ± 0.10 kcal·min^-1^) and P (0.99 ± 0.09 kcal·min^-1^) for the 3-hour period. Although no difference in energy expenditure was seen in the first hour, significant differences between SUP and P were observed in the second (1.10 ± 0.11 kcal·min^-1 ^and 0.99 ± 0.09 kcal·min^-1^, respectively; p = 0.02) and third hour (1.08 ± 0.11 kcal·min^-1 ^and 0.99 ± 0.09 kcal·min^-1^, respectively; p = 0.05). Average systolic BP was significantly higher (p = 0.007) for SUP (110.0 ± 3.9 mmHg) compared to P (107.3 ± 4.4 mmHg). No differences were seen in HR, diastolic BP, or POMS at any time point.

**Conclusions:**

Results showed a significant increase in energy expenditure in young, healthy women following an acute ingestion of a high-energy drink.

## Introduction

The overweight and obese population in America has been steadily increasing for the past 20 years [1,2]. This is suggested to be related to changes in the quality, quantity, source of food consumed, and a decrease in levels of physical activity in Americans [3]. Increased body fat and body weight are not only problematic for health reasons, but also lead to a decrease in self-esteem and have detrimental effects on mood [4]. This epidemic has caused many overweight and obese Americans to consume dietary supplements to aid in weight loss. The desire to reduce or control body fat appears to be one of the primary reasons for the consumption of energy drinks [5-7].

Energy drinks have been shown, or have been suggested, to increase alertness, energy, and to counteract fatigue [8,9]. In addition, energy drinks have been shown to favorably affect specific physiological functions such as lipolysis, substrate utilization, and energy expenditure [10]. As a result many manufacturers of energy drinks promote their products as effective agents in reducing body fat, and they believe that energy drinks should be part of weight loss programs.

Energy drink supplements often contain several herbal and botanical compounds that work synergistically to enhance energy expenditure, reduce body fat, and possibly enhance mood [11]. Caffeine is generally the basic ingredient in many energy drinks, and has been shown to be effective in enhancing lipolysis and fat oxidation [12,13]. When combined with other herbal compounds and thermogenic agents, caffeine has also been shown to increase alertness and concentration, improve mood, and reduce fatigue [9,14,15].

Despite the high frequency of energy drink consumption, limited studies are available to ascertain the efficacy of these drinks in young adult populations. In addition, with the vast amount of energy drinks that are available on the market containing varying ingredients it becomes difficult to extrapolate the results of a few studies on the benefits of all energy drinks. Thus, it becomes imperative to examine specific combinations of ingredients that are presently available in these drinks and determine the specific efficacy as it relates to the products contents. The purpose of this study is to examine the acute effects of a ready to drink (RTD) supplement marketed as Meltdown RTD™ on resting oxygen consumption, respiratory quotient, caloric expenditure, heart rate, blood pressure and mood in healthy, physically active women.

## Methods

### Subjects

Ten healthy, physically active women (20.4 ± 0.7 y; 166.9 ± 7.2 cm; 67.0 ± 7.0 kg; 29.6 ± 6.5% body fat; BMI 23.9 ± 3.5 kg/m^2^) underwent two testing sessions administered in a randomized and double-blind fashion. Subjects were recruited from The College of New Jersey through announcements in the Health and Exercise Science Department. Following an explanation of all procedures, risks, and benefits associated with the experimental protocol, each subject gave her written consent prior to participating in this study. Subjects completed a medical history and physical activity questionnaire to determine eligibility. Subjects who were pregnant, smokers, or taking regular medication except birth control pills were excluded from the study. Subjects with any known metabolic or cardiovascular disease, or psychiatric disorder were also excluded. Subjects were also required to have been free of any nutritional supplements or ergogenic aids for 6 weeks preceding the study, and were asked to refrain from taking any additional supplement during the course of the study.

### Study Design

The study followed a double-blind, crossover design. Subjects reported to the Human Performance Laboratory at the same time of day on two separate days. Each testing session was separated by an average of 7 days (7.0 ± 2.8 d). Subjects were instructed to refrain from consuming any caffeine products on the day of each testing session and from performing any strenuous physical activity for the previous 12 hours. In addition, subjects were instructed to be at least 3 hours post-absorptive state prior to each trial. During each visit to the laboratory, upon completing a 30 min resting period, subjects were randomly provided either (140 ml) of the energy drink supplement (SUP), commercially marketed as Meltdown RTD^® ^(Vital Pharmaceuticals, Inc., Davie, FL) or an equal amount of placebo (P) as suggested by the manufacturer's serving recommendation (two 70 ml servings separated by 30 minutes). On the subject's second visit to the laboratory they were provided with the opposite treatment. Measurement began immediately after the initial 70 ml consumption of SUP or P. A half hour following initial ingestion, measurement was paused as the subjects consumed the remaining 70 ml, and restarted immediately following consumption. Subjects remained resting in a semi-recumbent position during the entire measurement period. They were permitted to read, study, and/or listen to music following consumption and refrained from speaking during the study period. The total time of measurement was 3 hours. Oxygen consumption (VO_2_) and heart rate (HR) were determined every 5 min during the first 30 min and every 10 min during the next 150 min. Blood pressure (BP) was determined every 15 min during the first 30 min and every 30 min thereafter. The profile of mood states (POMS), and a questionnaire focusing on alertness, focus and fatigue were assessed every 30 min.

### Metabolic Measures

Immediately following supplement ingestion subjects were fitted with a Medgraphics preVent™ pneumotach (Medical Graphics Corporation, St. Paul, MN) to measure VO_2 _and respiratory quotient (RQ) through open-circuit spirometry using a metabolic measurement cart (CPX Ultima™ series, Medical Graphics Corporation, St. Paul, MN). Machine calibration was performed prior to each session using a 3-liter syringe and calibration gases of known concentration of oxygen and carbon dioxide. Measures of VO_2_, RQ, energy expenditure, and fat oxidation rate were obtained continuously for the entire 3-hour trial beginning one minute following supplement or placebo consumption. The average for every 5 min was recorded for the first 30 min, and every 10 min thereafter until 180 min post consumption. HR was also measured at these time points using a wireless HR monitor (Pacer, Polar CIC, Inc., Port Washington, NY), which updated HR display every 5 s. BP was measured using a sphygmomanometer and ausculatory method at 15 min and 30 min post ingestion, and then for every 30 min until data collection concluded.

### Questionnaires

The POMS was administered 7 times during each testing session. The initial POMS administration was given as the subject reported to the Human Performance Laboratory, and every half hour for the three hour period following supplement ingestion. All questionnaires were performed under controlled conditions (a quiet room alone with the investigator) and the same researcher performed all test administrations.

The POMS consists of 65 words or phrases in a Likert format questionnaire which provides measures of specific mood states. It provides measures of tension, depression, anger, vigor, fatigue and confusion. A total mood score is computed by subtracting vigor from the sum of the five other negative measures and adding 100 to avoid a negative result. McNair et al. [16], has reported internal consistency of measures ranging between 0.85 to 0.95 and test-retest reliability estimates ranging between 0.65 to 0.74. These lower coefficients of stability are thought to be indicative of transient and fluctuating characteristics of mood states. During all test administrations participants were asked to describe their feelings upon how they were feeling at that moment.

Subjects were also asked to complete an energy level questionnaire containing four questions using a 5-point rating scale. Subjects were asked to rate their energy level, fatigue level, feelings of alertness and feelings of focus for task using the following verbal anchors: 1 = very low; 2 = low; 3 = average; 4 = high; 5 = very high. The same researcher performed all test administrations and tests were conducted under controlled conditions (a quiet room).

### Supplement

On each visit subjects ingested either 140 ml of Meltdown RTD^® ^or a placebo. Meltdown RTD^® ^contains 230 mg of anhydrous caffeine, and the following herbal and botanical compounds; methyl tetradecylthioacetic acid, yerba mate extract, methyl-synephrine, methylphenylethylene, 11-hydroxy yohimbine, yohimbine HCL, alpha-yohimbine, and methyl-hordenine HCL. The placebo was similar in appearance and taste to Meltdown RTD^®^, but contained only an inert substance.

### Statistical Analyses

The area under curve (AUC) for VO_2 _was calculated by using a standard trapezoidal technique. RQ, HR, BP and POMS were averaged over each hour and the entire 180-minute period. Statistical analysis of the data was accomplished using a repeated measures analysis of variance. In the event of a significant F- ratio, LSD post-hoc tests were used for pairwise comparisons. AUC analysis and 180-minute comparisons were analyzed using dependent T-tests. A criterion alpha level of p ≤ 0.05 was used to determine statistical significance. All data are reported as mean ± SD. A sample size of 10 subjects provided 88% statistical power at an α level of 0.05 (two-tailed).

## Results

**Figure 1:**
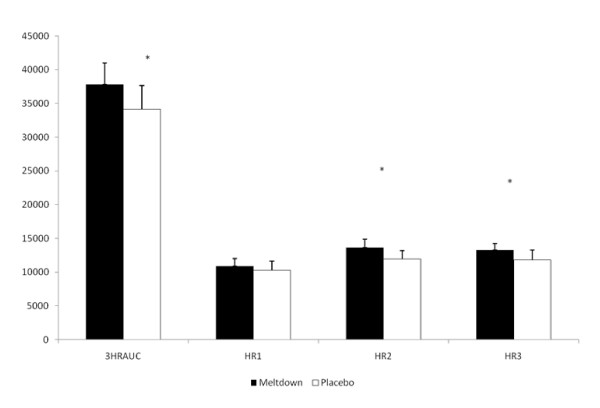
**Oxygen Consumption-AUC**. *P < 0.05, between groups; 3HRAUC = 3-hour average AUC; HR1 = 1^st ^hour; HR2 = 2^nd ^hour; HR3 = 3^rd ^hour.

**Figure 2:**
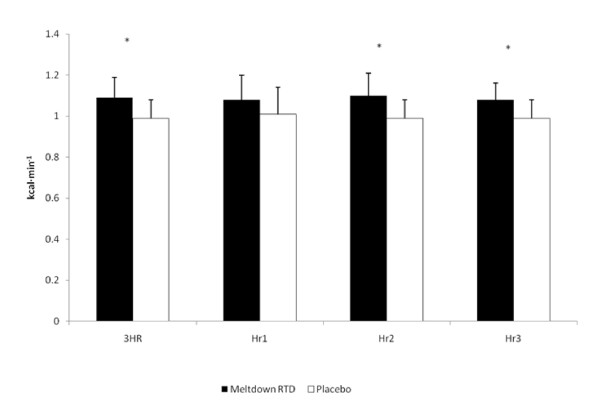
**Caloric Expenditure**. *P < 0.05, between groups; 3HRAUC = 3-hour average AUC; HR1 = 1^st ^hour; HR2 = 2^nd ^hour; HR3 = 3^rd ^hour.

The AUC analysis revealed a 10.8% difference (p = 0.03) in VO_2 _between SUP and P for the three hour study period. No significant differences in resting oxygen consumption were seen in the first hour following ingestion of the supplement. However, oxygen consumption was significantly elevated within the second hour (13.9%; p = 0.01) and third hour (11.9%; p = 0.03) following ingestion (Figure 1). The average hourly energy expenditure response to the study protocol is depicted in Figure 2. A significant difference (p = 0.03) in energy expenditure was seen between SUP (1.09 ± 0.10 kcal·min^-1^) and P (0.99 ± 0.09 kcal·min^-1^) for the 3-hour period. Although energy expenditure was not significantly different between SUP and P in the first hour, significant differences between the groups were seen in the second hour (1.10 ± 0.11 kcal·min^-1 ^and 0.99 ± 0.09 kcal·min^-1^, respectively; p = 0.02) and third hour (1.08 ± 0.11 kcal·min^-1 ^and 0.99 ± 0.09 kcal·min^-1^, respectively; p = 0.05). No significant change from resting values of RQ were seen between either SUP (0.86 ± 0.05) or P (0.88 ± 0.06) in the 3-hour study period.

Blood pressure and heart rate responses to the study protocol are displayed in Table 1. Significantly higher (p = 0.007) systolic BP (p < 0.01) was observed between SUP (110.0 ± 3.9 mmHg) and P (107.3 ± 4.4 mmHg) during the three hour study period. No change from resting levels and no significant differences were seen between the groups in HR or diastolic BP at any time point.

**Table 1:** Average Hourly Cardiovascular Measures

Variable		Baseline	Hour 1	Hour 2	Hour 3
Heart Rate (b·min^-1^)	SUP	77.7 ± 9.6	77.2 ± 10.3	76.8 ± 9.8	79.0 ± 9.4
	
	P	79.0 ± 11.6	80.6 ± 12.6	78.1 ± 10.5	78.7 ± 11.9

Systolic Blood Pressure (mmHg)	SUP	110.0 ± 3.9 *	110.2 ± 4.1 *	112.2 ± 4.9 *	109.1 ± 4.3 *
	
	P	107.3 ± 4.4	108.0 ± 5.0	106.4 ± 5.2	106.0 ± 4.0

Diastolic Blood Pressure (mmHg)	SUP	75.1 ± 3.2	75.5 ± 4.0	75.2 ± 3.6	74.9 ± 3.1
	
	P	74.6 ± 3.7	74.1 ± 4.6	73.1 ± 4.9	74.4 ± 3.9

*P < 0.05, between groups; SUP = Supplement; P = Placebo

**Figure 3:**
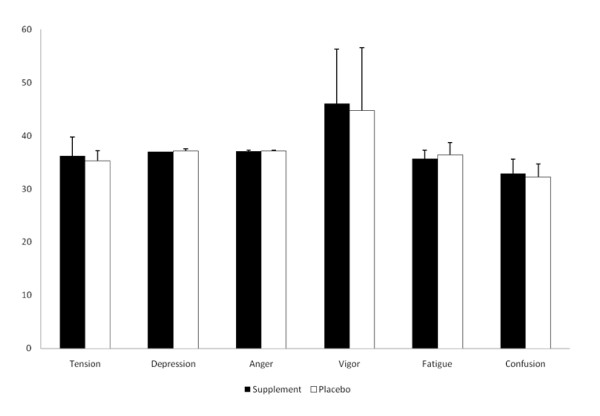
**Profile of Mood States**.

No significant differences were seen between SUP and P in any of the mood states measured during the study (Figure 3). In addition, no change from resting levels in perceived energy (3.0 ± 0.4 and 3.2 ± 0.6, respectively), fatigue (2.4 ± 0.5 and 2.5 ± 0.9, respectively), alertness (3.2 ± 0.3 and 3.2 ± 0.4, respectively), or focus (2.9 ± 0.4 and 3.2 ± 0.5, respectively) was noted between SUP and P during the three hour study period.

## Discussion

Results of this study support previous research indicating that the high-energy supplement Meltdown™ can enhance metabolic rate in healthy, college-aged students [17-20]. Prior studies examined the capsule form of the supplement, whereas this was the first examination of the liquid formulation. Similar to these investigations, ingestion of this supplement significantly increased systolic BP, but values remained within the normal range. In addition, no changes were noted to heart rate or diastolic BP indicating that this supplement may not pose a significant cardiovascular risk in apparently healthy subjects.

Previous studies have shown increases in resting energy expenditure over 2 to 3-hour time periods following caffeine ingestion in combination with additional ingredients including ephedra, black tea, green tea extract, *Citrus aurantium*, and yohimbine [17-23]. The common ingredient found in these studies has been caffeine, which has been shown to be very effective in elevating energy expenditure. Caffeine is thought to increase sympathetic nervous system activity by stimulating the alpha- and beta-adrenoreceptors, and to induce adenosine antagonism, which can lead to increase BP and heart rate [22,23].

The combination of caffeine and other herbal ingredients examined in this study resulted in a 10.8% increase in resting energy expenditure over the 3-hour period. The increase in energy expenditure for SUP was observed primarily during the second and third hour after consumption. Due to the manufacturer's serving recommendation, subjects consumed half (70 ml) of the supplement at baseline, and the remaining 70 ml after 30 minutes. It is likely that the thermogenic effect of the energy drink was delayed as a result of the ingestion pattern employed. These results also confirm previous findings by others that showed a delayed thermogenic effect from energy drink consumption for 30 - 60 minutes following ingestion, and then an ability to increase energy expenditure for the subsequent 120 minutes [8]. In addition, the results of this study also show a lower thermogenic effect (~18% difference) of this particular supplement compared to a previous study from our laboratory that examined the capsule form of this supplement [19]. It is possible that mode of ingestion (liquid versus capsule), stability differences between the two modes, or ingestion pattern (entire supplement ingested immediately or a staggered ingestion), may have contributed to the reduced thermogenic response. The staggered ingestion pattern likely limited the thermogenic response in the first hour of measurement.

The increase in the metabolic rate is consistent with other investigations examining caffeine and additional thermogenic agents [17-21,24-26]. In addition, the elevated systolic BP seen during the SUP treatment is also similar to other studies examining weight loss supplements containing adrenergic amines [21,25,27]. Synephrine, an active component of *Citrus aurantium*, has an adrenergic effect that may result in appetite suppression, increased metabolic rate, and lipolysis [28]. Alpha and beta-adrenergic drugs have well-documented effects on the cardiovascular system, indicating synephrine would have similar hemodynamic effects [28]. Similar to our findings, Hofstetter and colleagues [29] reported that synephrine intake in healthy subjects caused significant elevations in systolic and mean arterial pressures, while diastolic BP and heart rate were unchanged.

The findings of this study do not support previous research from our laboratory that indicated that the combination of yohimbine, yerba mate extract and tetradecylthioacetic acid can enhance utilization of stored fat [19]. Others have demonstrated that these ingredients, either by themselves or in combination, are effective in enhancing lipid metabolism [19,30-34]. The effect of this combination of ingredients has been realized following 60 minutes after ingestion of the capsule form of this supplement. It is possible that the staggered consumption pattern may have limited the effectiveness of this supplement regarding fat oxidation within the time frame examined. Yohimbine's effect may have also been modulated by its various metabolites within the supplement. Considering that there are differences in the α-2 adrenoceptor blocking potency and half-life between the metabolites of yohimbine [35], the staggered consumption pattern may have limited the ability to effectively generate a sufficient stimulus for enhanced lipolysis.

The addition of phenylethylamine as an ingredient was thought to enhance the mood of subjects using this supplement. Prior research has shown that phenylethylamine can produce relief of depression among a clinical population, even in those that were unresponsive to standard treatments [36]. The mechanism of action is thought to be related to the stimulation of dopamine release [37], which can not only improve mood, but may has also been shown to reduce appetite [38]. An advantage in the use of phenylethylamine is thought to be related to the beneficial improvements seen in mood without producing a tolerance often associated with amphetamines [36]. The results of this study however, do not support any role for phenylethylamine in affecting mood in apparently healthy, college-aged females consuming an energy drink. In addition, no difference in alertness, focus and energy was noted. However, this may have been a function of the passive nature of this study. Previous studies have shown that adrenergic stimulators are effective in enhancing alertness and focus in this age group performing high intensity activity [9].

In conclusion, the results of this study indicate that acute ingestion of Meltdown RTD™ increases energy expenditure in young, healthy women. In addition, ingestion of this supplement increases systolic blood pressure for three hours following ingestion. However, BP values stayed will within the normal range. No changes in mood, subjective feelings of alertness, focus, or energy were seen. Future research should examine the potential role that chronic intake of this supplement has on fat loss and body composition changes.

## Competing interests

Vital Pharmaceuticals. (Davie, FL) provided funding for this project. All researchers involved independently collected, analyzed, and interpreted the results from this study and have no financial interests concerning the outcome of this investigation. Publication of these findings should not be viewed as endorsement by the investigators, The College of New Jersey or the editorial board of the Lipids in Health and Disease.

## Authors' contributions

JRH obtained grant funds for project, designed study, supervised all study recruitment, data/specimen analysis, statistical analysis and manuscript preparation. SLR was the lead author, performed data collection and data analysis. JK, NAR, AC and ADF were co-authors, oversaw all aspects of study including recruitment, data/specimen analysis, and manuscript preparation. All authors have read and approved the final manuscript.
